# Development of Body Mass Index Growth Standard Chart for Chinese Preschool Children

**Published:** 2019-09

**Authors:** Chunjing TU, Chongmin JIANG, Sanhua ZHANG, Yan-Feng ZHANG, Rui CAI, Dongming WU, Huan WANG, Xiaoming HAN, Botong XU

**Affiliations:** 1. Qianjiang College, Hangzhou Normal University, Hangzhou, China; 2. China Institute of Sport Science, Beijing, China; 3. Department of Preschool Education, College of Education, Hangzhou Normal University, Hangzhou, China

**Keywords:** Preschool children, Lambda-median-sigma method, Body mass index, Growth chart, Growth standards, China

## Abstract

**Background:**

We aimed to establish a reference standard of Body Mass Index (BMI) for the growth of preschool children in China.

**Methods:**

We monitored and obtained the height and weight of 50702 children aged 3–6 yr in 31 provinces in mainland China in 2014. The reference standard and percentile curve of BMI preschool children aged 3–6 yr old were formulated by using Lambda-Median-Sigma (LMS) method in China.

**Results:**

The common grounds of the male and female children were as follows: the percentile maps were similar in shape; the graphs of children aged 4–6 were approximately horn shaped. The differences between male and female children were as follows: the BMI values of male children in the same age group and the same percentile were higher than those of the female children. The change pattern of male children was larger than that of female children. BMI of 3 yr old and 6 yr old children was larger than those of 4 yr old and 5 yr old. During the change from low percentile to high percentile, the BMI values of Chinese male children exceeded WHO to a larger extent, and the BMI values of Chinese female children were substantially consistent with WHO, but the high percentile greater than P95 exceeded WHO.

**Conclusion:**

The BMI growth chart developed can be applied in monitoring the growth and nutrition of preschool children in China. We recommend the promotion of the results in the field of preventive health care.

## Introduction

Overweight and obesity in children are increasing in many countries and regions (1), and children’s health has been increasingly gaining attention. According to a new study led by Imperial College London and WHO, the number of obese children and adolescents aged between 5 and 19 has increased tenfold in the past 40 years, especially in Asian countries (2). Since 1978, with the rapid development of China’s economy, the problem of children obesity has become more prominent, and the overweight and obesity rates continue to increase in Chinese preschool children (3, 4). At present, the age of the research objects for overweight and obesity in children is constantly moving downward.

To evaluate children’s growth and development, many international organizations and countries have developed Body Mass Index (BMI) percentile reference standards and growth curves involving preschool children aged 3–6, such as the BMI percentile standards formulated by WHO (5), CDC (6), and International Obesity Working Group (IOTF) (7). In recent years, relevant researches have been carried out in China, (8, 9), which used data from the 2005 Survey of physical development of children in nine cities in China to develop the growth curves of children and adolescents. Many scholars have developed the growth curves of 3–6 yr old children in a single city (10–13). However, the sample populations of these studies are from regional or individual cities. So far, a reference standard for the growth of preschool children that can represent the whole country in China has not been established.

The China Physical Fitness Surveillance Center has conducted four national surveys for people aged 3–69 yr old since 2000 to monitor the health of Chinese nationals. Based on this data, the present study used the large sample survey data of 3–6 yr old children in 31 provinces (municipalities and autonomous regions) in 2014 to develop the percentile reference standard and Z score reference standard and percentile growth curves for BMI-for-age of preschool children aged 3–6 in China, so as to assess the growth and development of preschool children in China and provide reference for prevention of overweight and obesity.

## Materials and Methods

The data was obtained from the 2014 national sample survey, conducted by the National Physical Fitness Surveillance Center. The survey followed the principle of stratified sampling.

First, all 31 provinces (autonomous regions and municipalities directly under the central government) in mainland China were selected. Second, three prefecture-level cities were randomly selected in the provinces under the jurisdiction of the provinces, considering the economic and social development levels and regional characteristics. Third, each prefecture-level city was divided into two urban and rural regions (Urban children refer to those whose parents have urban-registered residence and who live in the city; rural children refer to those whose parents have rural-registered residence and who live in rural areas), and test kindergartens were randomly selected in the areas under their jurisdiction. They were grouped according to gender and age. Children born in the same year were included in one sampling group, with a total of 16 groups. One hundred children were sampled from each age group, and 1,600 children were expected to be sampled from each province. Finally, an actual valid data of 50,702 cases (25390 males and 25312 females) were collected nationwide.

Written informed consent was obtained from Child guardian of each participant. The study protocol was approved by General administration of sport of China, Ministry of health of China and other ten participating institutes [NO. (2014–5)].

### Collection of data

The test time was from April to August of 2014, and the test equipment was designated and uniformly distributed by the National Physical Fitness Surveillance Center (Jianmin II, Beijing). All investigators and research staffs underwent one-week training sessions on use of the standardized protocols and instruments for data collection. The height was accurately 0.1 cm, and the weight was accurately 0.1 kg. BMI was calculated by dividing the weight (kg) by the square of height (cm). The body of children aged 3–5 yr old (37–72 months) were in a rapid development stage, their height and weight changed rapidly.

The test data recording methods were as follows: for children aged 3–5 yr old, 0.5 yr old was the group interval, and children aged 6 yr old were taken as a single group. The age month and the sample size of each group are shown in Table 1. All investigators and research staffs underwent one-week training sessions on use of the standardized protocols and instruments for data collection A stringent quality assurance and quality control program was implemented to ensure data validity and reliability.

**Table 1:** Three to 6 years old preschool children descriptive statistics of BMI

***Age (yr)***	***Age (Month)***	***Boys***				***Girls***			
		***n***	***X̅***	***SD***	***Skewness***	***n***	***X̅***	***SD***	***Skewness***
3.0	37–42	2002	15.95	1.74	1.50	2057	15.66	1.59	1.04
3.5	43–48	4313	15.80	1.58	1.67	4192	15.55	1.48	1.04
4.0	49–54	3179	15.75	1.52	1.36	3299	15.42	1.57	1.69
4.5	55–60	3240	15.71	1.70	1.94	3174	15.38	1.59	1.33
5.0	61–66	3521	15.74	1.82	1.48	3614	15.33	1.67	1.61
5.5	67–72	2964	15.79	1.92	1.62	2806	15.40	1.92	2.36
6.0	73–84	6171	15.96	2.15	1.60	6170	15.42	1.84	1.26

### Statistical analysis

The LMS method was used to construct a BMI-for-age reference standard for children aged 3–6 yr old. The LMS method was initiated by the British scholar Cole (7, 14, 15). In this method, the data with skewness are transformed into an approximately standard orthodox distribution by using Box-Cox power transformation (Lambda), so that the parameter L smooth curve is constructed, as well as the construction parameter median (Median) smooth curve and variation coefficient (Sigma) smooth curve, and thus the reference standard is obtained. For the calculation formula, see Cole’s literature (15). The specific steps are as follows:

#### ① Calculating L, M and S

Examples of calculating the parameters (Table 2). Eleven BMI values are given in the first column. The second column gives the corresponding natural logarithms of BMI and column 3 the reciprocals of weight (i.e. 1/weight). Beneath each column is the mean and SD of the eleven values.

**Table 2:** A worked example of the LMS method based on eleven BMI values

***Transform***	***None***	***Logarithmic***	***Reciprocal***	***L***
***Power***	***1***	***0***	***−1***	***2.10369***
	16.72	2.81661	0.05981	
	12.58	2.53211	0.07949	
	15.88	2.76506	0.06297	
	12.64	2.53687	0.07911	
	11.74	2.46300	0.08518	
	14.78	2.69327	0.06766	
	12.56	2.53052	0.07962	
	15.29	2.72720	0.06540	
	16.79	2.82078	0.05956	
	15.10	2.71469	0.06623	
	16.94	2.82968	0.05903	
**Mean**	14.63818	2.67544	0.069460	
**SD**	1.93417	0.13538	0.00957	
**M**	14.63818 (Ma)	14.51867(Mg)	14.39667(Mh)	14.76731(M)
**CV: S**	0.13322(Sa)	0.13538(Sg)	0.13898(Sh)	0.13241(S)

Annotation:
Ma=14.63818, Mg=e^2.67544^=14.51867, Mh=1/0.069460=14.39667; Sa=14.63818/1.93417, Sg=2.67544/0.13538, Sh=0.06946/0.00957; A= log(Sa/ Sh), B= log(Sa•Sh/ Sg^2^); L= - A/(2B)=2.10369; S= Sg•exp(AL/4)=0.13241; M= Mg+(Ma- Mh)L/2+ (Ma- 2 Mg+ Mh)L^2^/2=14.76731.

The transformations used in columns I to 3 each correspond to a particular power transform. Finally, the parameters L, M and S of this group of data are: L=2.10369, M=14.76731; S=0.13241.

#### ② Smooth curves of each age group parameter were drawn, and the functional equation of the parameter curves were constructed.

The determination coefficients R2 and mean-square error (MSE) of the linear, quadratic, cubic, exponential, and power fitting curves of each parameter of LMS were calculated. The fitting curves were compared according to the goodness of fit and the least square method. The fitting curve with larger R2 and smaller MSE was selected as the final curve, and the functional equation of the selected curve was constructed.

#### ③ The functional equation of the percentile of each age group was calculated.

The fitting curve functions of L(x), M(x), S(x) were substituted into C100a = M(x)(1+ L(x)S(x)Za)1/L(t) to obtain the functional equation of the percentile reference standard. Zα is the normal dispersion of the tail area α (for example, when the 97th percentile α = 0.97, Zα=1.88), and C100α is the percentile BMI value corresponding to Zα.

#### ④ Reference standards were developed, and percentile curves were drawn.

Any percentile or Z score reference standard was obtained according to (3), and the percentile curve was directly drawn using EXCEL.

#### ⑤ Accuracy test of reference standard.

The goodness of fit test was performed on the percentile of the original data and the percentile value obtained by the LMS method.

## Results

### Basic information of sample data

According to the data in Table 1, the sample size, mean value, standard deviation, and skewness of male and female BMI is seen. The interval coefficient range of male groups was between 1.36 and 2.15, and the interval was 1.04 to 2.36 in female groups. The standard value of Skewness of normal distribution is Zero, the BMI of each age group of male and female children is skewed, and both are right-biased. Therefore, using the LMS method to convert right-biased data into data that is approximately positively distributed is necessary. In order to eliminate the influence of abnormal data on the model, the data which was too high or too low was cleared before the model was built. This study refers to Cole TJ’s literature (7), which deleted the sample data on height, weight, and BMI beyond M±5s. Overall, 35 cases (0.14%) and 29 cases (0.11%) in male and female were deleted, respectively. The number of samples used for modeling was 25329 and 25283, respectively.

### Development of BMI-for-age growth charts Construction of parametric equations

According to the determination coefficient (R^2^), mean square error (MSE), and smoothness of curves, the optimal fitting curve was selected, and the parameter equations of each BMI age group L(x), M(x), and S(x) were calculated using the LMS method. When the original data were collected, we divided each half-year age group from 3 to 5.5 yr old, and each one-year age group from 6 yr old. For the consistency of all age groups, Sample age “t” was converted into Parameter“x”.

The relationship between the parameter “x” and the age “*t”* in the formula is:

$$\text{x}=\{\begin{array}{c} 4t-11\;\text{if}\;3\;\text{year}\leq t\leq 5.5\;\text{year}\\ 14\;\text{if}\;t=6\;year \end{array}.$$

**Fig. 1:**
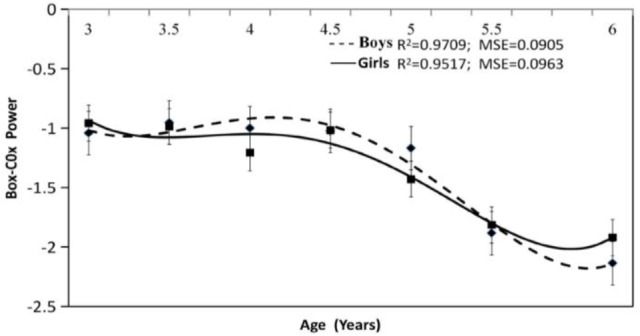
3 to 6 year preschool children L curve of BMI

① In the L(x) fitting curve (Fig. 1), the equation for male children was a quartic polynomial, and its determination coefficient was R^2^=0.97, MSE=0.09. The equation for female children was a quartic polynomial, and its determination coefficient was R^2^=0.95, MSE=0.01, the L curves of male and female children both had a desirable goodness of fit. The regression equations are as follows:

$$\begin{array}{l} \text{Lboy}\left(\text{x}\right)=0.00052269\;\;\;\;\text{x}4-0.01460704\\ \text{x}3+0.11717737\;\;\;x2-0.30684039\;\;\text{x}-0.8165757 \end{array}$$

$$\begin{array}{l} \text{Lgirs}\left(\text{x}\right)=0.00048264\;\;\;\;\;\;\;\;\;\;\;\;\;\;\text{x}4-0.0135039\\ \text{x}3+0.11466862\;\;\;\text{x}2-0.37277013\;\;\;\text{x}-0.66733602 \end{array}$$

**Fig. 2:**
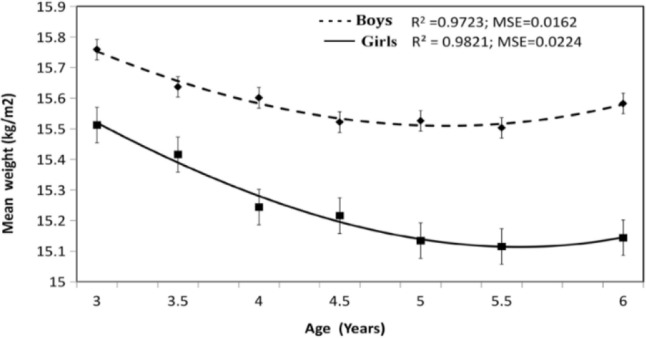
3 to 6 year preschool children M curve of BMI

② In the M(x) fitting curve (Fig. 2), the equation for male children was a cubic polynomial, and its determination coefficient was R^2^=0.97, MSE=0.02. The equation for female children was a cubic polynomial, and its determination coefficient R^2^=0.98, MSE=0.02, M curves of male and female children both had a desirable good ess of fit. The curves of male and female children are similar. In comparison, the curve of male children becomes gentle at 4.5 yr old and Bending upwards at 5.5 yr old, and the curve of female children becomes flat after 5 yr old without a remarkable change at 5–6 yr old. The M values of male children in all age groups were higher than those of female children, and the difference between male and female was statistically significant (*P*<0.01) (Fig. 2).

$$\begin{array}{l} \text{Mboy}\left(\text{x}\right)=0.00003289\;\;\;\;\;\;\;\;\;\;\;\;\;\;\text{x}3-0.0025939\\ \text{x}2-0.05918051\;\;\;\text{x}+15.80928882 \end{array}$$

$$\begin{array}{l} \text{Mgirl}\left(\text{x}\right)=0.00007567\;\;\;\;\;\;\;\;\;\;\;\;\;\;\text{x}3-0.00192935\;\;\;\;\text{x}2-0.073757\\ \text{x}+15.5910962 \end{array}$$

**Fig. 3:**
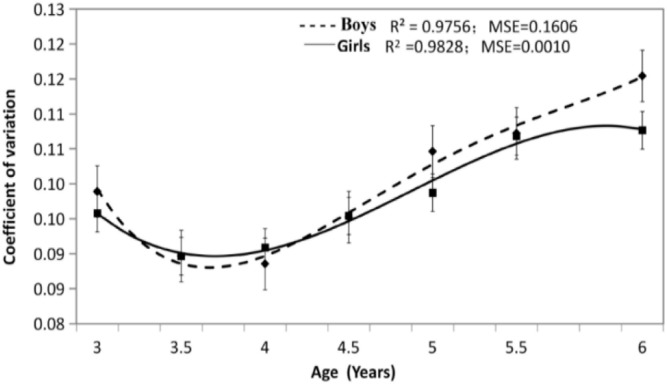
3 to 6 year preschool children S curve of BMI

③ In the S(x) fitting curve (Fig. 3), the equation for male children was a cubic polynomial, and its determination coefficient was R^2^=0.98, MSE=0.16. The equation for female children was a cubic polynomial, and its determination coefficient was R^2^=0.98, MSE=0.00, the modeling can achieve good results. As can be seen from (Fig. 3), the male and female curves are similar in shape, change slightly from 3 to 3.5 yr old, and gradually become larger after 4 yr old, with the increase of age, the number of overweight, obesity, or low weight may increase.

$$\begin{array}{l} \text{Sboy}\left(\text{x}\right)=-0.00005686\;\;\;\;\;\;\;\;\;\;\;\;\;\;\text{x}3+0.00152342\text{x}2-\\ 0.00959755\;\;\text{x}+0.10665788\\ \text{Sgirl}\left(\text{x}\right)=-0.0000454\;\text{x}3+0.00115691\;\text{x}2-\\ 0.00684218\;\text{x}+0.10143424 \end{array}$$

Finally, Substituting Lboy(x), Mboy(x), Sboy(x) and Lgirl(x), Mgirl(x), Sgirl(x) into C100a=M(x)(1+ L(x)S(x)Za)1/L(x), the functional equations of the percentile curve of BMI-for-age for male and female children can be obtained.

$$\begin{array}{l} \text{Boys}:\;\;\text{C}100\text{a}==\left(\left(3.29\text{e}-05\right)\;\;\text{x}3-\left(2.59\text{e}-03\right)\;\;\;\text{x}2-(5.92\text{e}-02)\;\text{x}+15.81\right)*\\ \{1+\left(\left(5.23\text{e}-04\right)\text{x}4\;-\left(1.46\text{e}-02\right)\text{x}3+0.12\text{x}2-0.31\text{x}-0.82\right)\;\;.(\left(5.69\text{e}-05\right)\text{x}3+\;\;\;\;\;\;\;\;\;\\ (1.52\text{e}-03)\text{x}2-\left(9.60\text{e}-03\right)\text{x}+0.11)*\text{Za}\}1/(5.23\text{e}-04)\text{x}4-\\ (1.46\text{e}-02)\text{x}3+0.12\text{x}2-0.31\text{x}-0.82\\ \text{Girls}:\text{C}100\text{a}==((7.57\text{e}-05)\;\;\;\text{x}3-(1.93\text{e}-03)\;\;\;\text{x}2-\left(7.38\text{e}-02\right)\;\;\;\text{x}+15.59)*\\ \{1+((4.83e-04)\text{x4}\;\;\;\;-(1.35\text{e}-02)\text{x}3+0.11\text{x2}-0.37\text{x}-0.67).((4.54\text{e}-05)\text{x}3+\\ (1.16\text{e}-03)\text{x2}-\left(6.84\text{e}-03\right)\text{x}+0.10)\;\;\;\;*Z\text{a}\}1/4.83\text{e}-04)\text{x4}-\left(1.35\text{e}-02\right)\text{x}3+0.11\text{x}2-0.37\text{x}-0.67 \end{array}$$

### Percentile reference standard and percentile curve

**Fig. 4:**
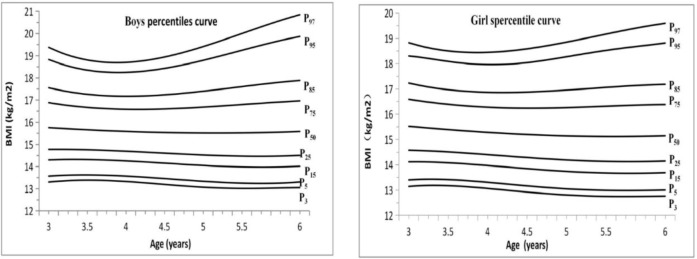
**(Left)** 3 to 6 year boys percentiles curve of BMI-for-age; **(Right):** 3 to 6 year girls percentiles curve of BMI-for-age

Substituting the correspondent Za value of percentile in the functional equation, we can directly yield the reference value of each percentile and its growth curve. As the fitting curves of L(x), M(x) and S(x) were all smooth curves, any one of the constructed percentile curves was smooth and did not need to be corrected again. According to the comparison between the fitted value and the actual value (Table 3), the accuracy of the male and female models has met the basic requirements. The error rates were low, and in particular the error rate of P50 was within 0.5%.And then we have the prediction growth chart (Table 4 and Fig. 4).

**Table 3:** Comparing fitted data with measured data of BMI in 3 to 6 years old preschool children percentile

***Sex***	***Age/Years***	***P_15_***			***P_50_***			***P_85_***		
		***Fitted data kg/m^2^***	***measured data kg/m^2^***	***rate of deviation (%)***	***Fitted data kg/m^2^***	***measured data kg/m^2^***	***rate of deviation(%)***	***Fitted data kg/m^2^***	***measured data kg/m^2^***	***rate of deviation (%)***
M	3.0	14.30	14.44	−1.00	15.76	15.76	0.00	17.55	17.30	1.50
	3.5	14.30	14.42	−0.80	15.64	15.61	0.20	17.25	17.10	0.80
	4.0	14.27	14.40	−0.90	15.60	15.58	0.20	17.19	17.03	0.90
	4.5	14.13	14.29	−1.10	15.52	15.46	0.40	17.21	17.03	1.00
	5.0	14.06	14.21	−1.10	15.53	15.42	0.70	17.40	17.29	0.60
	5.5	13.98	14.17	−1.40	15.50	15.41	0.60	17.61	17.44	1.00
	6.0	14.02	14.12	−0.80	15.58	15.48	0.70	17.88	17.94	−0.30
F	3.0	14.12	14.26	−1.05	15.52	15.46	0.38	17.22	17.09	0.80
	3.5	14.08	14.20	−0.84	15.39	15.41	−0.12	16.98	16.86	0.71
	4.0	13.97	14.02	−0.35	15.28	15.22	0.42	16.87	16.79	0.46
	4.5	13.85	13.97	−0.88	15.20	15.19	0.01	16.86	16.76	0.61
	5.0	13.74	13.91	−1.27	15.14	15.07	0.44	16.94	16.69	1.53
	5.5	13.68	13.76	−0.64	15.11	15.07	0.30	17.08	17.00	0.43
	6.0	13.69	13.78	−0.68	15.14	15.08	0.45	17.17	17.15	0.16

**Table 4:** 3 to 6 years old Preschool children percentiles and Z score of BMI-for-age

***Sex***	***Age (yr)***	***L***	***M***	***S***	***Percentiles (BMI in kg/m^2^)***									***Z Score***					

					***P3***	***P5***	***P15***	***P25***	***P50***	***P75***	***P85***	***P95***	***P97***	***-3sd***	***-2sd***	***-1sd***	***1sd***	***2sd***	***3sd***
Boys	3	−1.0203	15.7527	0.09853	13.3	13.6	14.3	14.8	15.8	16.9	17.6	18.8	19.4	12.2	13.2	14.3	17.5	19.6	22.4
	3.5	−1.0346	15.6560	0.09004	13.4	13.6	14.3	14.7	15.6	16.7	17.3	18.4	18.8	12.3	13.3	14.3	17.2	19.1	21.5
	4	−0.9205	15.5823	0.08965	13.3	13.6	14.3	14.7	15.6	16.6	17.2	18.3	18.7	12.3	13.2	14.3	17.1	19.0	21.3
	4.5	−0.9780	15.5334	0.09462	13.2	13.4	14.1	14.6	15.5	16.6	17.2	18.4	18.9	12.1	13.0	14.2	17.1	19.1	21.6
	5	−1.3059	15.5107	0.10223	13.1	13.3	14.1	14.5	15.5	16.7	17.4	18.8	19.4	12.0	13.0	14.1	17.3	19.7	23.0
	5.5	−1.8026	15.5159	0.10974	13.0	13.3	14.0	14.5	15.5	16.8	17.6	19.3	20.1	12.0	12.9	14.0	17.5	20.5	25.5
	6	−2.1475	15.5794	0.11487	13.1	13.3	14.0	14.5	15.6	17.0	17.9	19.9	20.8	12.0	12.9	14.1	17.8	21.4	29.2
Girls	3	−0.9385	15.5193	0.09570	13.1	13.4	14.1	14.6	15.5	16.6	17.2	18.4	18.9	12.0	13.0	14.2	17.2	19.2	21.7
	3.5	−1.0791	15.3892	0.09009	13.2	13.4	14.1	14.5	15.4	16.4	17.0	18.1	18.6	12.1	13.1	14.1	16.9	18.8	21.2
	4	−1.0508	15.2800	0.09047	13.1	13.3	14.0	14.4	15.3	16.3	16.9	18.0	18.4	12.0	12.9	14.0	16.8	18.7	21.0
	4.5	−1.1310	15.1953	0.09466	12.9	13.2	13.8	14.3	15.2	16.2	16.9	18.0	18.5	11.9	12.8	13.9	16.8	18.8	21.4
	5	−1.4119	15.1388	0.10047	12.8	13.0	13.7	14.2	15.1	16.3	16.9	18.3	18.9	11.8	12.7	13.8	16.9	19.2	22.4
	5.5	−1.8003	15.1140	0.10573	12.8	13.0	13.7	14.1	15.1	16.3	17.1	18.5	19.2	11.8	12.6	13.7	17.0	19.7	24.2
	6	−1.9247	15.1443	0.10782	12.8	13.0	13.7	14.1	15.1	16.4	17.2	18.8	19.6	11.8	12.6	13.7	17.1	20.0	25.1

We have the prediction growth chart (Fig. 4 and Table 4).From the growth chart, the similarities and differences of BMI between male and female children can be obtained. Specifically, ① the common features are as follows: BMI was similar in shape with the percentile graph with the increase of age, where the distance between P3 and P97 of 3–4 yr old was gradually reduced. The distance between P3 and P97 of 4–6 yr old gradually increased, and the percentile curve graph of 4–6 yr old was in horn shape. P50–P3 < P97–P50, and the graph was up-biased. For P97 and P95, which were far away from P50, the percentage change was large. P85 and P95 curves of male children bent upwards at about 4 yr old, and the curves of female children bent upwards at the age of 4.5. However, the low percentage change of P3, P5 was small; thus, no obvious turning point in the body thinning rate was observed. ② The different features are as follows: The distance between P3 and P97 of female children was smaller than that of male children, especially in 3 and 6 age groups. Comparing the BMI values of male and female in the same age group and the same percentile, the male values were all larger than those of female children, the overweight and obesity rate of female children is lower than that of male children.

## Discussion

### Percentile standard characteristics of this study

In general, the BMI values of male children in the same age group and the same percentile were higher than those of the female children, and the BMI percentile figures of male and female preschool children aged 3–6 were similar in shape. The right sides of the figure were similar in shape like a horn, and the opening of the figure of male children was larger than that of female children. From the specific percentile curve, male and female children of P3, P5, and P15 had slight change in each age group, and the percentile curves of P97 and P95 were far from P50 and changed fast, indicating that the rate of overweight and obesity increases with age after 4–6 yr old. In the P85, P95 or M+SD, and M+2SD standard deviation unit (Z score), male and female children were at the lowest position at 4–4.5 yr old. The reasons for gender differences may be related to differences in growth and development between male and female children and may also be accounted to the “preference of boys to girls” idea in China. The results of gender differences and percentile shape in this study are similar to those conducted in some cities of China (8, 11, 13).

### Comparison of Chinese percentile standards with WHO standards

**Fig. 5:**
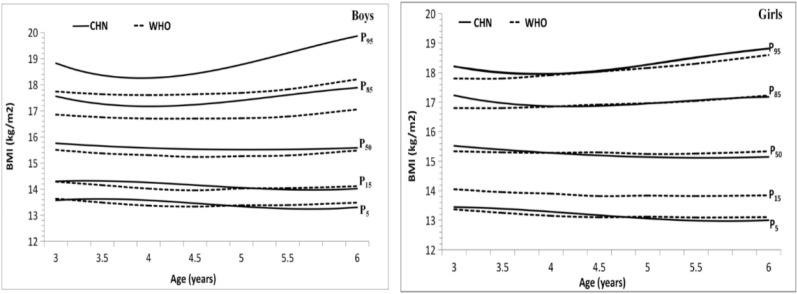
**(Left)** BMI percentile standard Comparison between China and WHO (boys); **(Right):** BMI percentile standard Comparison between China and WHO (girls)

Comparing the standard of this study with WHO standard (2006) (16) (Fig. 5), the opening amplitude of the left and right ends of the Chinese percentile graph is greater than that of WHO.

Specifically, ① Chinese male child BMI percentile was generally larger than the data from WHO, Chinese BMI values P5 and P15 were basically consistent with WHO data, and P50 was also consistent with WHO. P50 was 0.2–0.3 kg /m^2^ higher than WHO. P85 was 0.5–0.9 kg/m^2^ higher than WHO. P95 was 0.7–1.7 kg/m^2^ higher than WHO, in the change process from low percentile to high percentile, the percentile value of Chinese male children BMI exceeded WHO standard increasingly, especially in 3 and 6 age groups, which were at the left and right ends. ②BMI percentile of female children is generally larger than WHO, but the difference is small. The differences of male and female children were inconsistent with WHO. The result may be related to the fact that the gender difference of Chinese children’s body weight was different from that of the WHO sample population. The WHO data of children older than 5 yr old was from the United States, where the BMI in P95 female children was significantly higher than that of male children. By contrast, the value of female in China was lower than that of male.

The corresponding percentiles of pointcuts of WHO overweight and obesity (17) M+SD, M+2SD were P84.1, and P97.7, respectively. If the WHO reference standard is used to judge the rate of overweight and obesity of 3–6 yr old children in China, the rate of male children will far exceed the overweight and obesity rate of the WHO standard sample population, but the rate of female children is only slightly larger than that data. For example, the obesity pointcut value of WHO 6-year-old male children M+2SD was 18.8 kg/m^2^, and using the LMS inverse equation $\text{Z}=\frac{{\left(\frac{MI}{M}\right)}^{L}-1}{LS}$ , the Z score was first calculated. Then, the normal distribution table was checked to convert to the percentile. 18.8 kg/m^2^ was located in P91.0 in the percentile curve of this study and much smaller than P97.7. The BMI value of the 6-year-old female children had a pointcut value of 19.5 kg/m^2^, located in P97.3 in the percentile curve of the study, slightly lower than P97.7.

### Validity of the results of this study

The research objects are broadly representative. The data of this study comes from the national survey of Chinese national physique in 2014. The valid samples of 3–6 yr old are more than 50,000. It is the largest cross-sectional survey on growth and development in China, and the sample population is widely representative.

The LMS method was applied in various countries, such as the US CDC (6), International Obesity Organization Working Group (1OTF) (7, 19), and WHO (5), used this method to develop relevant growth criteria. In China, the LMS method has been applied in the development of growth reference standards, such as height, weight, BMI, blood pressure, waist circumference (9, 19, 20), and has been proven to be feasible in theory and practice.

## Conclusion

We successfully constructed BMI Growth Chart for Chinese Preschool Children, the difference between the fitted value and the actual value is small, and the curve smoothing effect is good. The BMI growth chart developed in this study can be applied in monitoring the growth and nutrition of preschool children in China. We recommend the promotion of the results in the field of preventive health care.

## Ethical considerations

Ethical issues (Including plagiarism, informed consent, misconduct, data fabrication and/or falsification, double publication and/or submission, redundancy, etc.) have been completely observed by the authors.
